# Good Clinical Approach: Delphi Consensus for the Use of Betahistine in Menière's Disease

**DOI:** 10.1155/2018/5359208

**Published:** 2018-10-25

**Authors:** Augusto Pietro Casani, Elena Navari, Giorgio Guidetti, Michel Lacour

**Affiliations:** ^1^Department of Surgery and Medicine, ENT Section, Pisa University Hospital, Italy; ^2^Vertigo Center PCM, Modena, Italy; ^3^Université Aix-Marseille University/CNRS, UMR 7260, Fédération de Recherche 3C, Centre de St Charles, Marseille, France

## Abstract

Menière's disease is a disorder of the inner ear that causes vertigo, tinnitus, fullness, and hearing loss. Several pharmacological treatments are available, but none of them has shown significant results. Betahistine has been largely used but its effect on the main symptoms of Menière's disease remains unclear. In order to improve clinical appropriateness and to reduce the heterogeneity of the therapeutic approaches for Menière's disease, we proposed a European Consensus Conference on Betahistine's prescription. A group of European experts in vestibular disorders completed a questionnaire, prepared by opinion leaders, on the use of betahistine in Menière's disease. The Delphi method was used as an iterative investigation method in order to increase and establish the consensus. While betahistine was considered useful to reduce the number of the vertigo attacks during the intercritical phase of the disease, its use during attacks was considered helpful only when associated with other drugs. Betahistine was not considered useful for preventing hearing loss. The experts support the use of betahistine during the intercritical phase of the disease to reduce the number and severity of vertigo episodes. They also defined the parameters for a good clinical approach to evaluate the efficacy of betahistine treatment for Menière's disease.

## 1. Introduction

Menière's disease (MD) is an idiopathic pathological condition of the inner ear (IE) whose major symptoms are vertigo, fluctuating hearing loss, tinnitus, and fullness [1]. These symptoms are most often unilateral, although the possible bilateral involvement of the IE does exist. Moreover, 75% of the patients with unilateral symptoms also showed bilateral endolymphatic hydrops (EH) [2]. Several studies showed an extreme heterogeneity of data, with percentages of IE involvement ranging from 2% to 78% of the patients [1, 3]. MD etiology is still under debate. Some authors believe that EH is a consequence of various factors such as genetic [4] and environmental [4, 5] factors. Others postulate that EH is the result of obstructions of the endolymph flow [6]. Moreover, some studies support the involvement of infections and immunologic and even psychological factors [6–8]. MD is frequently associated with other diseases, such as migraine [9] and autoimmune disease [10].

A US health database report showed a prevalence of 190 per 100,000 in MD [11]. However, in population-based studies, the prevalence fluctuates from 200 to 500 per 100,000 [12]. Although there is a discordance in the gender-distribution of the disease [13, 14], there is a general agreement that MD is a middle-age disease, and that its prevalence increases with age.

Given the high controversy for MD's etiology, treatment strategies are multiple and mostly empirical. Moreover, given the natural instability of the disease, which includes long remission phases, clinicians have difficulties to carry out Clinical Class A studies that would provide better indications for clinical practice [15].

Medical treatment for MD mostly depends on the disease phase and can be divided into medications for the acute attack treatment, and prophylactic treatments prescribed during the intercritical period, between the attacks.

During the acute attacks, antiemetic and vestibular suppressant drugs, such as benzodiazepines, antihistamines, anticholinergics, and antidopaminergics, are often used to control vertigo episodes [16]. Some of them have an additional beneficial anxiolytic effect. However, long-term administration may elicit adverse effects, such as nausea, vomiting, and impairment of vestibular compensation [16]. The possible autoimmune origin for MD has suggested the use of steroids, which can reduce the magnitude of the crisis and promote auditory and vestibular recovery [16]. Finally, osmotic diuretics (mannitol or glycerol 10%) might also be used as a treatment [16].

The prophylaxis treatment for the intercritical phase aims to reduce the number and severity of vertiginous crises, to relieve chronic symptoms (instability and tinnitus), and to prevent the progression of the disease, especially hearing loss and equilibrium disorders. The frequency and the severity of the crises may be reduced in 2/3 of patients controlling dietary regimen, use of caffeine, alcohol and nicotine. A control of stress, fatigue, and allergy may also contribute to minimize the symptoms [16, 17]. A pharmacological approach may include the use of diuretics, corticosteroids, vasodilators, or labyrinthine deafferentation with aminoglycosides [5, 16]. Also complementary and alternative medicine and rehabilitation therapies are used to treat MD patients [16]. Nevertheless, no effective therapy has shown real efficacy for long-term hearing preservation. In Europe, the most widely used drug to treat MD patients is betahistine, a histamine-like molecule [17]. The major effect of betahistine is in the management of vertigo. Both preclinical and clinical studies suggest a wide range of potential effects for betahistine. First, it modulates histaminergic neurotransmission by partially agonizing the activity of the histamine H1 receptor. Betahistine has however more potent antagonistic properties at the histamine H3 receptor [18], inducing an increased histamine turnover and release. Second, betahistine has vascular effects both in the cochlea and in the brain [18–22]. Third, betahistine has effects on neuronal excitability and spike generation of neurons in the lateral and medial vestibular nuclei. It has been recently demonstrated that histamine induces an excitatory modulation by depolarizing both spontaneous firing neurons and silent neurons in the rat inferior vestibular nucleus via the histamine H1 and H2 receptors [23]. It is possible that the betahistine antivertigo activity is firstly achieved by betahistine itself and then sustained by its metabolite aminoethyl pyridine [24, 25]. Finally, several studies and reviews indicate that the histamine H3 receptor plays a key role in vestibular compensation, behavioral recovery, and reduction of symptoms [26–29].

Taken together, these studies confirm the beneficial therapeutic effects of betahistine in MD and vestibular vertigo patients. However, the betahistine improvement on the whole MD symptoms is still controversial. The latest Cochrane review on the efficacy of betahistine in MD concluded that betahistine is acceptable to use; however no evidence of its efficacy has been really demonstrated [30]. In a meta-analysis evaluating clinical studies in patients with vertiginous symptoms, Della Pepa et al. confirmed the therapeutic benefit of betahistine [31]. More recently, Nauta supported also the therapeutic benefit of betahistine on vertiginous symptoms in both Menière's disease and vestibular vertigo patients [32]. But most of these studies were performed out of the gold clinical standards, and in addition some inconclusive findings might be attributable to different methodologies used in the analysis and data processing.

Most of the drugs, including betahistine, were developed several decades ago at a time when evaluative epidemiology was still emerging. Nowadays, the need of randomized double blind and placebo controlled clinical trials is necessary for a clear and adequate evaluation of their efficacy [31, 32]. Given the inconsistency reported in the literature and in order to define best practice criteria in MD therapy appropriateness, we proposed a European Consensus Conference on Betahistine's prescription for MD, using the Delphi method.

## 2. Methods

The Delphi method is an iterative investigation method that aims to reach an agreement and to provide recommendations among experts on a controversial topic [33, 34]. It is frequently used in healthcare, when group of experts in the field collect their opinions freely, individually and anonymously through a series of round of discussion. After each round, an administrator provides a summary of the experts' answers and their rationale. All the topics were discussed until an agreement was found among the participants to the consensus.

**Figure 1:**
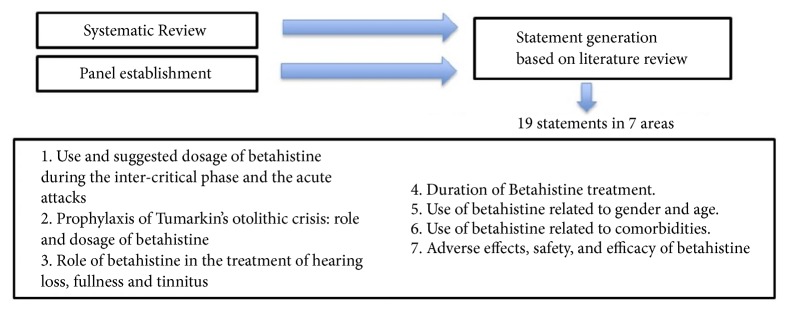
Process used to select and achieve consensus for statements about betahistine in Menière's Disease.

A group of opinion leaders, after formulating questions, selected articles to circulate to all participants, who reported their comments on a form. For each article, two experts prepared a number of statements, for which no agreement has been found in the literature, in order to be discussed. Based on clinical cases, randomized clinical trial, and controlled clinical trial recommendations for Menière Disease, the following topics were considered: dosage and length of treatment, disease phase treatments options, age/gender, comorbidity, and adverse effects (Figure 1).

A questionnaire made of 15 statements, each of them declined in 4 or more items, was then distributed among the selected experts who expressed their level of agreement according to the following 5-point scale: 1 = absolutely disagree, 2 = disagree, 3 = agree, 4 = more than agree, and 5 = absolutely agree. Consensus was reached when the sum of items 1 and 2, or 3, 4, and 5 reached 66%. A Consensus Conference (CC), with 80 European field experts, was then organized to discuss the result of the questionnaire and to obtain agreement for the responses on which a consensus or not was reached.

## 3. Results

A panel of 80 European experts (a list of the participants is provided in the appendix), from 10 different European Countries, received a questionnaire of 15 items on the use and efficacy of betahistine in Menière's disease. The results of the Delphi questionnaire were the base for the CC held in Rome in December 2015.

### 3.1. Use and Suggested Dosage of Betahistine during the Intercritical Phase and the Acute Attacks

Regarding the use of betahistine for the management of vertigo and dizziness during the intercritical phase of MD, the panel reached a full consensus for its usefulness (87% of agreeing answers) and as the first-choice drug (71% of agreeing answers) (Table 1).

Based on scientific evidences, the panel agreed that the dosage of betahistine for prophylaxis therapy may vary in the course of time (78% of agreeing answers) and advised a dosage between 32 and 48 mg/day (97% agreeing answers) (Table 1). During the attacks, 66% of the experts agreed that betahistine is not useful and shows poor efficacy (88% of agreeing answers) (Table 1). However, the panel agreed on the use of betahistine when associated with other therapies (72% of agreeing answers) (Table 1). When surveyed on the dosage for the acute attacks, no consensus was reached after the Delphi questionnaire. For this reason, a series of evidences were pointed out in order to reach a new consensus. The subsequent vote showed positive consensus (73%) among the participants regarding the poor efficacy of betahistine for the management of the acute attack and would not recommend a betahistine dosage between 32 and 48 mg/day (87% of negative consensus) (Table 2).

### 3.2. Prophylaxis of Tumarkin's Otolithic Crisis: Role and Dosage of Betahistine

In the management of Tumarkin's otolithic crisis, the drug has been evaluated as not efficacious (90% agreeing answers), and useful only if associated with other molecules (66% agreeing answers).

### 3.3. Role of Betahistine in the Treatment of Hearing Loss, Fullness and Tinnitus

Similarly, the efficacy of the drug is low in contrasting progressive auditory deterioration (81% agreeing answers); the participants considered betahistine not useful in therapy of fullness (67% agreeing answers) and useful only when associated with other drugs regarding the tinnitus symptoms (71% of agreeing answers). No agreement was obtained for each of the items about the hearing symptoms (Table 3).

### 3.4. Duration of Betahistine Treatment

The experts were also surveyed on the duration of the treatment based on the number of Menière's crises during the last 6-month time period. The experts agreed on a 3-month treatment in cases of 1-3 crises events (88% agreeing answers), extendable to 6 months, depending on patient's condition (96% of agreeing answers) (Table 4).

In cases of 4 to 10 crises in the last 6 months, the suggested duration of the treatment was 6 months (67% agreeing answers), extendable to 1 year, depending again on patient's condition (79% of agreeing answers) (Table 4).

Agreement regarding the duration of treatment for patients with more than 10 attacks during the reference period was at least three months (66% of agreeing answers), six months (89% of agreeing answers), and six months extendable to 1 year (90% of agreeing answers) (Table 5). No consensus was reached for more than one-year treatment option (Table 5).

### 3.5. Use of Betahistine Related to Gender and Age

The panel agreed that betahistine efficacy depends neither on the gender nor on the age of the MD patients (Table 6). However, no consensus was reached, when surveyed on specific age ranges (Table 6, items 4, 5, and 6).

### 3.6. Use of Betahistine Related to Comorbidities

When MD is associated with migraine, the experts agreed to use betahistine (69% of negative consensus on the items 1 and 3), but they favored comedication only in association with antimigraine drugs (86% agreeing answers). In cases of comorbidity with anxious-depressive disorders, betahistine was suggested to be used in combination with antidepressant or anxiolytic drugs only (93% agreeing answers) (Table 7).

### 3.7. Adverse Effects, Safety, and Efficacy of Betahistine

Based on their professional experience, the panel defined gastric disorders as the most common betahistine adverse effects (90% of agreeing answers). The experts considered also betahistine as a safe drug, with a 10% chance of side effects (91% agreeing answers) (Table 8).

As a conclusion of the CC, the experts defined a list of parameters for evaluation of betahistine efficacy in treatment of MD.  Table 9 shows a positive consensus for number of attacks in the last 6 months, intensity or severity of attacks (Vertigo score), quality of life, days of work/number of days being disabled in the last 6 months, frequency of attacks (1 year), questionnaire on social impact of Menière disease, and questionnaire DHI (100%, 98%, 96%, 76%, 79%, 95%, 79%, and 93% of agreeing answers, respectively). Negative consensus was reached for tinnitus evaluation (76% of disagreeing answers). Finally, no consensus was found for six out of fifteen items proposed (Table 9).

## 4. Discussion

Menière disease is a disorder of the IE associated with a wide range of various symptoms. It is still difficult to treat this pathology given the lack of knowledge on the etiopathogenetic factors and the few number of gold standard clinical trials (class A studies) [15]. To date, the pharmacological management of MD remains heterogeneous, and it mostly depends on the empirical experience of the medical doctors, which differ from one country to another.

In Europe, the most frequently prescribed drug in the treatment of MD is betahistine, a weak H1 agonist and strong H3 antagonist histaminergic molecule [18]. Betahistine targets different systems (central nervous system, vascular tree) and has a dose-dependent effect on neuronal excitability [23, 24, 35], cochlear and cerebral blood flow [18–21]. In MD patients, betahistine reduces the frequency and severity of MD attacks [36]. Interestingly, betahistine exerts a key role on vestibular compensation, given its action at the H3 histamine receptor, favoring recovery and reduction of the residual symptoms in both animal models [37] and unilateral vestibular loss patients [38, 39].

Betahistine efficacy in MD management is still controversial and inconsistent. Although the most recent Cochrane Review has concluded that betahistine is an acceptable treatment [40], the authors did not find strong evidences for the efficacy of the drug in the management of dizziness, tinnitus, loss of hearing, or ear fullness. Moreover, the meta-analysis carried out by Della Pepa et al. [31] reported ambiguous evidence on the role of the drug in the management of the symptoms associated with dizziness, but it questioned the methodological approaches used in the different clinical studies [31].

Given the controversial data available in the literature and the high heterogeneity in the MD therapeutic prescriptions, the Delphi method was used as a way to provide a better clinical approach to MD treatment based on a consensus on efficacy, safety, and treatment duration elaborated by experts in the field. The following represents the discussion of the results obtained during the CC.

As a main result of the CC, the experts agreed that betahistine is the first choice of drug and is really useful during the intercritical phase therapy for the management of vertigo in MD patients, with a dosage from 32 to 48mg/day (or varying in the course of time according to the patient's condition). During the attacks, all the experts agreed that betahistine is not useful alone, but that it can be used positively in association with other drugs. The appropriate dosage of betahistine in combined therapies is also 32 to 48 mg/day, with the option of variable doses depending on the patient's condition. It was highlighted that if the attack occurred in patients already under betahistine treatment, increasing the dosage of betahistine may be useful in these cases. However, based on the literature reports, a more efficacious and faster effect of antiemetics and vestibular suppressant drugs was recognized by the experts. Since betahistine improves vestibular compensation but is known to be rapidly metabolized, a different administration route of the drug should be a way to increase its efficacy [41]. Regarding the prophylaxis of Tumarkin's otolithic crisis, there is negative consensus for the usefulness of betahistine at any dosage. Higher levels of anxiety and the onset of hearing loss in the asymptomatic ear have been found in patients experiencing otolithic crisis [42]. It would be more appropriate to establish a psychiatric and psychological evaluation to decide of the therapeutic approach rather than to use betahistine for Tumarkin's otolithic patients [42].

A contrasting topic was for the use of betahistine in hearing symptoms therapy. After discussion, the participants agreed only on the usefulness of betahistine in controlling the hearing symptoms in the advanced stage of the disease. No consensus was reached for a possible role of betahistine in the early phase or in the improvement of the disease development. This is most likely due to the different approaches that the European physicians have about hearing symptoms.

There has been a general agreement on the duration of betahistine treatment, according to the number of attacks during the last six months. For patients with 1-3 attacks in the last six months, the general agreement was at least a three months treatment, extendable to six months depending on patient's condition. When the number of attacks raised up to 4-10 during the same reference period, the experts recommended at least a six months treatment, with a possible extension up to one year. Finally, for MD patients having more than 10 attacks during the last six months, experts suggested a therapy lasting from three months up to one year. Extension of the therapy for more than one year did not get consensus due to the lack of supporting studies on the long term, especially regarding safety issue.

MD is often diagnosed in association with other pathologies, such as migraine and depression [7, 9, 11]. The panel participants agreed for the association of betahistine with antimigraine or anxiolytic and/or antidepressant drugs in such cases. This is also beneficial given the limitation for daily activities that MD patients face. When surveyed on the adverse effects, all the experts agreed on betahistine as a safe drug with a frequency of adverse effects lower than 10%. Among the side effects they highlighted gastric disorders as the most common. Moreover, they confirmed the efficacy of betahistine, regardless of age and gender, as described previously [18].

Based on their daily clinical practice and considering that the main use of betahistine is to relieve MD patients of their vertigo episodes, the experts considered the number and intensity of attacks, clinical vestibular examinations, and questionnaires on quality of life (QoL), as the best parameters or tools to evaluate betahistine efficacy. They gave a negative consensus tinnitus, hearing symptoms, audiometric tests and instrumental vestibular examinations, and psychometric questionnaires. This view is most probably due to the lack of evidences for betahistine's ability to reduce tinnitus and contrast results from trials performed so far [17]. Moreover, clinical trials for MD are mainly limited by the certainty of the diagnosis in the patients recruited and the comorbidity conditions. Likewise, the lack of completed databases and patient registries may be a limiting factor in evaluating betahistine efficacy on MD symptoms.

Based on the evidences in the literature and the discussion during the CC, the experts listed the following parameters for evaluating the efficacy of betahistine in MD management:

- Number of attacks for 6 months
- Intensity or severity of attacks (Vertigo score)
- Quality of life questionnaires
- Frequency of attacks (1 year)
- Questionnaire DHI
- Intensity of attacks in last 6 months
- Questionnaire on social impact of Menière's disease
- Days of work/number of days being disabled

The only negative consensus was reached for tinnitus evaluation. Many other parameters did not reach any consensus, including audiogram, bed-side examination, caloric test, and evaluation of eye movement (nystagmus). In fact, there is very few or even no reliable data evaluating betahistine efficacy on eye movement and hearing symptoms.

## 5. Conclusions

A Delphi method has been used to find an agreement on the role of betahistine in current management of MD. The experts reached a clear consensus on the following:

- Betahistine is the first-choice drug in the intercritical phase therapy for the management of vertigo in MD patients (99%) with a dosage that should range from 32 to 48 mg/day (74%), with the possibility to vary the dosage during the time-course of treatment (76%);
- In case of 1-3 attacks in the last six months, treatment with betahistine needs to be at least 3 months (88%), extendable to six months depending on the patient's condition (96%);
- Treatment with betahistine should be at least of 6 months extendable to 1 year, depending on the patient's condition, in case of 4-10 attacks in the last six months (79%) or in case of more than 10 attacks (90%);
- The best results are obtained independently on ages (80%) and gender.
- In case of comorbidity, betahistine can be used in association either with antimigraine (86%) or anxiolytic and/or antidepressant (93%) drugs;
- Adverse effects of betahistine are less than 10% (91%);
- The most common side effects are gastric disorders (90%);
- Number, intensity and duration of attacks, bed-side vestibular examination, and questionnaire on QoL are the best ways to evaluate betahistine efficacy (100%).
- In conclusion, the participants to the Consensus Conference, based on their clinical practice and personal experience with their patients, support betahistine's prescription for MD to reduce the number and severity of dizziness crises, particularly during the intercritical phase of the disease.

## Figures and Tables

**Table 1:** Results of the Consensus Conference on the use and dosage of betahistine during the intercritical phase of MD. 1 = absolutely disagree, 2 = disagree, 3 = agree, 4 = more than agree, and 5 = absolutely agree. Consensus was reached when the sum of items 1 and 2 or 3, 4, and 5 reached 66%. Asterisk explanations:  ^*∗*^***Negative consensus***,  ^*∗∗*^***No consensus***, and  ^*∗∗∗*^***Positive consensus***.

	1	2	3	4	5

***In the inter-critical phase therapy, referred to the vertigo in the Menière's disease, do you think that betahistine:***					

**1. **Is useful	8%	5%	41%	23%	23%

	**13**%		**87**% ^*∗∗∗*^		

**2. **Is useful only when associated to other therapies	8%	66%	12%	12%	2%

	**74**% ^*∗*^		**26**%		

**3. **It's the first-choice drug	3%	26%	19%	26%	26%

	**29**%		**71**% ^*∗∗∗*^		

**4. **Shows poor efficacy	49%	45%	2%	4%	0%

	**94**% ^*∗*^		**6**%		

***If you use Betahistine for prophylaxis what is the dosage that you use:***					

**1. **I do not use	45%	36%	6%	3%	10%

	**81**% ^*∗*^		**19**%		

**2. **I use in the range from 8 to 32 mg/day	21%	64%	9%	3%	3%

	**85**% ^*∗*^		**15**%		

**3. **I use in the range from 32 to 48 mg/day	3%	0%	41%	29%	17%

	**3**%		**97**% ^*∗∗∗*^		

**4. **I use higher than 48 mg/day	4%	31%	50%	6%	9%

	**35**% ^*∗∗*^		**65**% ^*∗∗*^		

**5. **Vary in the course of time	3%	19%	54%	8%	16%

	**22**%		**78**% ^*∗∗∗*^		

**Table 2:** Results of the Consensus Conference on the use and dosage of betahistine during the acute attacks of MD. 1 = absolutely disagree, 2 = disagree, 3 = agree, 4 = more than agree, and 5 = absolutely agree. Consensus was reached when the sum of items 1 and 2 or 3, 4, and 5 reached 66%. Same asterisk convention.

***During an attack, referred to the vertigo in the Menière's disease do you think that betahistine:***					

**1. **Is the first-choice drug in monotherapy	47%	27%	16%	5%	5%

	**74**% ^*∗*^		**26**%		

**2. **Is useful when associated to other therapies	14%	14%	47%	23%	2%

	**28**%		**72**% ^*∗∗∗*^		

**3. **Is not useful	17%	17%	46%	3%	17%

	**34**%		**66**% ^*∗∗∗*^		

**4. **Shows poor efficacy	9%	3%	48%	9%	31%

	**12**%		**88**% ^*∗∗∗*^		

***If you use Betahistine during acute attacks, what is the dosage?***					

**1. **I do not use betahistine during acute attacks	10 %	17 %	34 %	19 %	20 %

	**27**%		**73**% ^*∗∗∗*^		

**2. **Range from 8 to 32 mg/day	44 %	43 %	13 %	0 %	0 %

	**87**% ^*∗*^		**13**%		

**3. **Range from 32 to 48 mg/day	19 %	28 %	38 %	9 %	6 %

	**47**% ^*∗∗*^		**53**% ^*∗∗*^		

**4. **Higher than 48 mg/day	30 %	20 %	34 %	13 %	3 %

	**50**% ^*∗∗*^		**50**% ^*∗∗*^		

**Table 3:** Results of the Consensus Conference on the role of betahistine in the therapy of long lasting hearing loss, fullness and tinnitus. 1 = absolutely disagree, 2 = disagree, 3 = agree, 4 = more than agree, and 5 = absolutely agree. Consensus was reached when the sum of items 1 and 2 or 3, 4, and 5 reached 66%. Same asterisk convention.

	1	2	3	4	5

***In the therapy of hearing loss in the Menière's disease, do you think that betahistine:***					

**1. **Is the first-choice drug in monotherapy	31%	29%	33%	0%	7%

	**60**% ^*∗∗*^		**40**% ^*∗∗*^		

**2. **Is useful when associated to other therapies in the early stages and in the advanced phases of the disease	18%	22%	50%	4%	6%

	**40**% ^*∗∗*^		**60**% ^*∗∗*^		

**3. **Is useful when associated to other therapies ONLY in the early stages of the disease	11%	26%	44%	14%	5%

	**37**% ^*∗∗*^		**63**% ^*∗∗*^		

**4. **Shows poor efficacy	2%	17%	52%	12%	17%

	**19**%		**81**% ^*∗∗∗*^		

***In the therapy of tinnitus in the Menière's disease, do you think that betahistine:***					

**1. **Is the first-choice drug in monotherapy	13%	50%	26%	7%	4%

	**63**% ^*∗∗*^		**37**% ^*∗∗*^		

**2. **Is useful when associated to other therapies	6%	23%	51%	13%	7%

	**29**%		**71**% ^*∗∗∗*^		

**3. **Is not useful	10%	53%	27%	7%	3%

	**63**% ^*∗∗*^		**37**% ^*∗∗*^		

**4. **Shows poor efficacy	7%	34%	46%	7%	6%

	**41**% ^*∗∗*^		**59**% ^*∗∗*^		

**Table 4:** Results of the Consensus Conference on betahistine treatment duration. 1 = absolutely disagree, 2 = disagree, 3 = agree, 4 = more than agree, and 5 = absolutely agree. Consensus was reached when the sum of items 1 and 2 or 3, 4, and 5 reached 66%. Same asterisk convention.

	1	2	3	4	5

***For how long do you think it is useful to extend treatment in case of more than 1-3 attacks in the last six months:***					

**41. **Less than 3 months	22%	50%	25%	0%	3%

	**72**% ^*∗*^		**28**%		

**42. **At least 3 months	0%	12%	76%	8%	4%

	**12**%		**88**% ^*∗∗∗*^		

**43. **At least 3 months, extendable to six months depending on the patient's condition	0%	4%	69%	11%	16%

	**4**%		**96**% ^*∗∗∗*^		

**44. **More than 6 months	6%	29%	51%	6%	8%

	**35**% ^*∗∗*^		**65**% ^*∗∗*^		

***For how long do you think it is useful to extend treatment in case of more than 4-10 attacks in the last six months:***					

**47. **At least 3 months	23%	40%	23%	9%	6%

	**63**% ^*∗∗*^		**37**% ^*∗∗*^		

**48. **At least 6 months	6%	27%	30%	19%	19%

	**33**%		**67**% ^*∗∗∗*^		

**49. **At least 6 months, extendable to 1 year depending on the patient's condition	3%	18%	29%	21%	29%

	**21**%		**79**% ^*∗∗∗*^		

**50. **More than 1 year	6%	49%	29%	9%	9%

	**54**% ^*∗∗*^		**46**% ^*∗∗*^		

**Table 5:** Results of the Consensus Conference on betahistine treatment duration. 1 = absolutely disagree, 2 = disagree, 3 = agree, 4 = more than agree, and 5 = absolutely agree. Consensus was reached when the sum of items 1 and 2 or 3, 4, and 5 reached 66%. Same asterisk convention.

***For how long do you think it is useful to extend treatment in case of more than 10 attacks in the last six months:***					

**53. **At least 3 months	9%	25%	40%	5%	21%

	**34**%		**66**% ^*∗∗∗*^		

**54. **At least 6 months	0%	11%	50%	25%	14%

	**11**%		**89**% ^*∗∗∗*^		

**55. **At least 6 months, extendable to 1 year depending on the patient's condition	5%	5%	50%	7%	33%

	**10**%		**90**% ^*∗∗∗*^		

**56. **More than one year	0%	38%	44%	11%	7%

	**38**% ^*∗∗*^		**62**% ^*∗∗*^		

**Table 6:** Results of the Consensus Conference on the use of betahistine related to gender and age. 1 = absolutely disagree, 2 = disagree, 3 = agree, 4 = more than agree, and 5 = absolutely agree. Consensus was reached when the sum of items 1 and 2 or 3, 4, and 5 reached 66%. Same asterisk convention.

	1	2	3	4	5

***You think that the best results can be obtained:***					

**1. **In a female patient	14%	59%	24%	0%	3%

	**73**% ^*∗*^		**27**%		

**2. **In a male patient	14%	63%	19%	3%	1%

	**77**% ^*∗*^		**23**%		

**3. **In a patient younger than 20	9%	69%	17%	3%	3%

	**77**% ^*∗*^		**23**%		

**4. **In patients aged 20 - 40	9%	53%	29%	9%	1%

	**61**% ^*∗∗*^		**39**% ^*∗∗*^		

**5. **In patients aged 41 - 65	7%	49%	36%	1%	7%

	**56**% ^*∗∗*^		**44**% ^*∗∗*^		

**6. **In patients older than 65	9%	52%	26%	7%	6%

	**61**% ^*∗∗*^		**39**% ^*∗∗*^		

**7. **At all ages, indifferently	4%	16%	29%	26%	26%

	**20**%		**80**% ^*∗∗∗*^		

**Table 7:** Results of the Consensus Conference on the use of betahistine in comorbidity cases. 1 = absolutely disagree, 2 = disagree, 3 = agree, 4 = more than agree, and 5 = absolutely agree. Consensus was reached when the sum of items 1 and 2 or 3, 4, and 5 reached 66%. Same asterisk convention.

	1	2	3	4	5

***In case of comorbid migraine:***					

**1. **I do not use betahistine	21%	47%	23%	4%	4%

	**69**% ^*∗*^		**31**%		

**2. **I use an association of betahistine plus an antimigraine drug	1%	13%	47%	20%	19%

	**14**%		**86**% ^*∗∗∗*^		

**3. **I use only betahistine	25%	66%	9%	0%	0%

	**91**% ^*∗*^		9%		

***In case of comorbid anxiety and/or depression:***					

**4. **I do not use betahistine	33%	51%	9%	4%	3%

	**84**% ^*∗*^		**16**%		

**5. **I use an association of betahistine plus an anxiolytic and/or antidepressant drug	4%	3%	49%	23%	21%

	**7**%		**93**% ^*∗∗∗*^		

**6. **I use only Betahistine	37%	51%	10%	1%	1%

	**88**% ^*∗*^		**12**%		

**Table 8:** Results of the Consensus Conference on the adverse effects, safety, and efficacy of betahistine. 1 = absolutely disagree, 2 = disagree, 3 = agree, 4 = more than agree, and 5 = absolutely agree. Consensus was reached when the sum of items 1 and 2 or 3, 4, and 5 reached 66%. Same asterisk convention.

	1	2	3	4	5

***In my experience, the following side effects are more common:***					

**1. **Headache	21%	60%	16%	3%	0%

	**81**% ^*∗*^		**19**%		

**2. **Insomnia	26%	67%	3%	4%	0%

	**93**% ^*∗*^		**7**%		

**3**. Gastric disorders	*1*%	*9*%	*51*%	*28*%	*11*%

	**10**%		**90**% ^*∗∗∗*^		

**4. **Asthmatic crises	16%	62%	19%	3%	0%

	**78**% ^*∗*^		**22**%		

**5. **Diarrhea	17%	61%	19%	3%	0%

	**78**% ^*∗*^		**22**%		

**6. **Sexual dysfunctions	33%	66%	1%	0%	0%

	**99**% ^*∗*^		**1**%		

**7. **Sedation	29%	48%	20%	3%	0%

	**77**% ^*∗*^		**23**%		

***You think that the adverse effects of betahistine are:***					

1. Less than 10%	4%	5%	24%	27%	40%

	**9**%		**91**% ^*∗∗∗*^		

2. From 10 to 20%	31%	55%	10%	3%	1%

	**86**% ^*∗*^		**14**%		

3. More than 20%	51%	45%	3%	0%	1%

	**96**% ^*∗*^		**4**%		

**Table 9:** Results of the Consensus Conference on the parameters to be used to evaluate betahistine efficacy. 1 = absolutely disagree, 2 = disagree, 3 = agree, 4 = more than agree, and 5 = absolutely agree. Consensus was reached when the sum of items 1 and 2 or 3, 4, and 5 reached 66%. Same asterisk convention.

	1	2	3	4	5

***In your opinion, what are the best parameters of efficacy to evaluate usage of Betahistine in the Ménière's disease treatment?***					

Intensity or severity of attacks (vertigo score)	2%	0%	40%	17%	41%

	**2**%		**98**% ^*∗∗∗*^		

Number of attacks for 6 months	0%	0%	36%	19%	45%

	**0**%		**100**% ^*∗∗∗*^		

Quality of life questionnaires	0%	4%	55%	11%	30%

	**4**%		**96**% ^*∗∗∗*^		

Days of work/number of days being disabled	2%	22%	60%	8%	8%

	**24**%		**76**% ^*∗∗∗*^		

Intensity of attacks in last 6 moths	4%	17%	52%	5%	22%

	**21**%		**79**% ^*∗∗∗*^		

Questionnaire on social impact of Meniere disease	3%	18%	50%	12%	17%

	**21**%		**79**% ^*∗∗∗*^		

Questionnaire DHI	2%	5%	60%	10%	23%

	**7**%		**93**% ^*∗∗∗*^		

Audiogram	17%	31%	41%	4%	7%

	**48**% ^*∗∗*^		**52**% ^*∗∗*^		

## Data Availability

The data used to support the findings of this study are included within the article.

## Appendix

List of the participants to the Consensus Conference

***From Italy***Asprella Libonati Giacinto, Balzanelli Cristiano, Barbara Maurizio, Barbara Michele, Boninsegna Marco, Bozzini Isabella, Califano Luigi, Cambò Michele, Carbonaro Vito, Castellucci Andrea, Cerchiai Niccolò, Chiarella Giuseppe, Chiarelli Raffaele, Coppo Gianfranco, Cozzolino Giovanni, Damiani Sergio, Degli Innocenti Massimo, Del Colle Raffaele, Falco Carlo Emilio, Frau Nicolò, Gamba Paolo, Gino Roberto, Latini Gino, Lucisano Sergio, Mandara Mario, Manzari Leonardo, Melucci Walter, Mininni Sebastiano, Navari Elena, Palmieri Giampaolo, Pintucci Jeanpierre, Pollastrini Lanfranco, Portaleone Sara Maria, Rosignuolo Mario, Scarpone Roberto, Scotto Di Santillo Leonardo, Spadon Marko, Stagno Rosetta, and Tringali Giuseppe.

***From Spain*** Aleman Lopez Oscar, Aran Gonzalez Ismael, Benitez Rosario Jesus, Espinosa Sanchez Juan Manuel, Fraile Rodrigo Jesus, Martin Sanz Eduardo, Oliva Dominguez Manuel, Perez Garrigues Herminio, Perez Vazquez Maria Paz, and Trinidad Ruiz Gabriel.*** From Poland*** Grazyna Tacikowska, Katarzyna Pierchata, and Malgorzata Jagielska Kubiczek.*** From Portugal*** Araujo Pedro Alexandre Espirito Santo Matias, Castillo Rosa Maria, Coneicao Valadas Monteiro Maria, Costa Redondo Cancela Amorim Ana Margarida, Da Silva Cunha Nuno Maria Trigueiros, Duarte Costa Sandra Marisa, Furtado Soares Tomè Pedro Augusto, Leonel Luis, Pires Mendes Santos Henriques Maria Manuel, Rosmaninho Seabra Josè Carlos, Soares Martins Joao Gabriel, and Vaz Garcia Fernando.*** From Romania*** Albu Silviu Anghel Daniela, Cozma Sebastian, Marceanu Luigi, and Roceanu Adina.*** From France*** Robin Philippe.
